# Antibodies in Aseptic Meningitis of Connective Tissue Disorder: A Case Report

**DOI:** 10.7759/cureus.60762

**Published:** 2024-05-21

**Authors:** Sreevinishaa Ravichandran, J Kumar, Nirmala Devi Chandrasekaran, Shahul Irfan, Sathvika A

**Affiliations:** 1 General Medicine, Sri Ramasamy Memorial (SRM) Medical College Hospital and Research Centre, Chennai, IND; 2 Internal Medicine, Government Medical College and Hospital Cuddalore, Chidambaram, IND; 3 Internal Medicine, Sri Ramasamy Memorial (SRM) Medical College Hospital and Research Centre, Chennai, IND

**Keywords:** systemic lupus erythematosis, anti-u1rnp autoantibody, cns manifestations, mixed connective tissue disorder, aseptic meningitis

## Abstract

Mixed connective tissue disorder (MCTD) is the first overlap syndrome described with features of overlapping manifestations of at least two other autoimmune rheumatic conditions. It is an autoimmune disease of rarity and is strongly associated with specific antibodies to U1 small nuclear ribonucleoprotein (anti-U1-RNP). This disorder affects almost all organs of the body, and it has varied clinical presentations as it has an autoimmune and inflammatory background, causing heightened immune cell activation. They present more commonly with less fatal symptoms like joint pain, stiffness, and mucocutaneous changes. The majority present initially with Raynaud's phenomenon followed by muscular skeletal involvement and around half of them present with swallowing problems due to esophageal dysmotility. Rarely do they also present with more morbid symptoms of pulmonary hypertension and central nervous system involvement. MCTD on follow-up had a 10 percent association with neurological manifestations as reported by the National Organization for Rare Diseases (NORD), and the most reported diseases were trigeminal neuralgia and aseptic meningitis. Patients presenting with such symptoms and, when treated only with guideline-based antibiotics therapy, would delay the treatment, leading to a poorer prognosis. The following is an interesting case of a young female presenting with a headache, which was masquerading as an underlying undiagnosed connective tissue disorder. Headache is a predominant presentation that has several etiologies in autoimmune disease and meticulous differential diagnosis workup is a must. This case highlights the fact that any persistent atypical, unusual symptom needs to be always considered for further evaluation to arrive at a diagnosis and for a favorable outcome.

## Introduction

Mixed connective tissue disorder (MCTD) is a disorder with overlapping symptoms of other autoimmune diseases like Systemic Lupus Erythematosus (SLE), scleroderma, rheumatoid arthritis, polymyositis, and dermatomyositis and is associated with U1-RNP which are antibodies targeted to the U1 small nuclear ribonucleoprotein (RNP) autoantigen. The neurological manifestations in mixed connective disease disorder are quite rare with an incidence of 10 to 17% [1,2] and range from acute psychosis, trigeminal neuralgia, coma, and subacute dementia to chronic illness. On the other hand, SLE as a separate entity has a greater incidence of neurological presentations. This difference in central nervous system (CNS) manifestations is either due to the genetic makeup of the patient or the heightened immune response is still a subject of perplexity. The genetic response is related to the human U1-RNP which is a long RNA molecule with seven Sm proteins and three proteins 70K, A, and C in U1 particles [3]. In MCTD, immune cell activation leads to modification of the RNP antigen, and the subsequent generation of B cells and T cells leads to the formation of these autoantibodies [4,5]. The exact role of these autoantibodies in the causation of neurological manifestations is ill-defined. The rarity of CNS manifestations in MCTD and the clinical importance of these antibodies are highlighted in this case report.

## Case presentation

An 18-year-old female presented to the emergency department with an altered sensorium of one day duration with a preceding history of low-grade fever for 20 days associated with headache and vomiting. There was no significant family or travel history, no recent history of vaccination, head injury, or contact with tuberculosis. There was a history of pain and swelling of small joints of the hands and feet on and off for the past one year. The patient had no complaints of early morning joint stiffness, but the mother had noticed some abnormal behavior and seizure-like activity, for which the patient didn’t seek proper medical attention or further evaluation. There was a history of discoloration of fingers on exposure to cold. On examination, the patient was drowsy, responding to painful stimuli. Consent was obtained from the attendant, who was the patient's mother and we proceeded with the examination. Her Glasgow coma scale (GCS) was 11/15 with an eye-opening of 3, verbal response of 2, and motor response of 6 (E3V2M6). Her vitals were stable with oxygen saturation of 97 percent in room air, temperature of 102 degrees F and capillary blood glucose level of 100 mg/dl.

On detailed CNS examination, neck stiffness was found present, and Kernig’s sign was positive. Both pupils were equal and reacting to light. The plantar was flexor on both limbs. The tone and power of both upper and lower limbs were normal. The sensory system and cerebellum could not be examined. There were no skin changes or rash. There was swelling of small joints of hands and feet with no associated redness. Other system examinations were normal. A provisional diagnosis of acute febrile illness with altered sensorium with differential diagnosis of acute demyelinating encephalomyelitis and viral meningoencephalitis was made. For a patient with fever, headache, or altered sensorium with a background history suggestive of some connective tissue disorder, a differential diagnosis of both infectious and non-infectious causes should be suspected.

**Figure 1:**
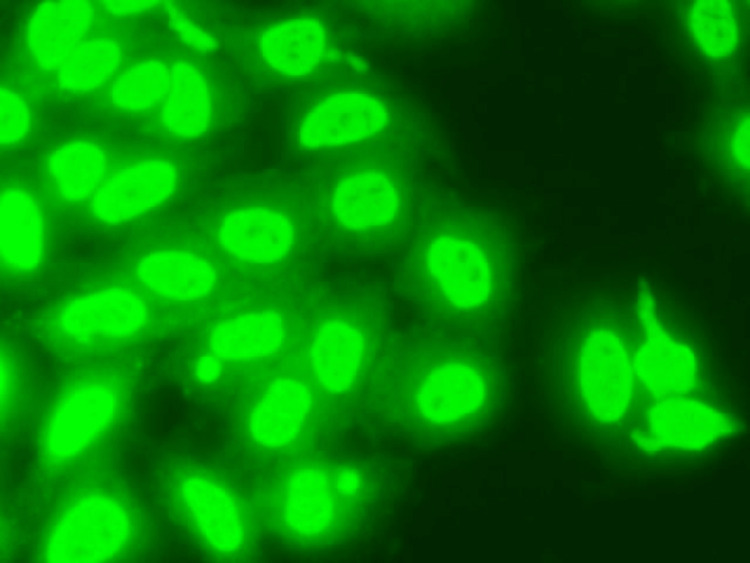
Immunofluorescence image The image shows a coarse speckled pattern of anti-nuclear antibody (ANA).

Her hemogram, renal and liver function tests, serum electrolytes, and fever serology profile, which covered dengue, scrub typhus, enteric fever, and leptospirosis were normal. C-reactive protein level was 95.2 mg/l. Serum angiotensin-converting enzyme level was 22 U/L (normal values are 12-68 U/L), and antinuclear antibody (ANA) titers were strongly positive ++++(1:10000) with a speckled pattern (Figure 1).

**Figure 2:**
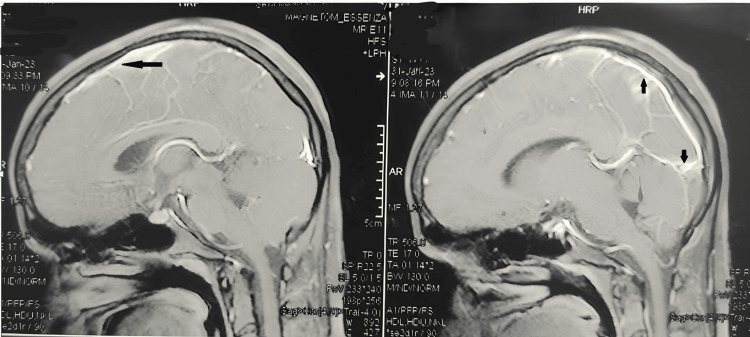
Contrast-enhanced MRI brain images Leptomeningeal enhancement is seen in the images (black arrows).

The antibody to U1-RNP/Sm was strongly positive by immunoblot assay. Anti-neutrophil cytoplasmic antibodies were negative. Rheumatoid factor (RF) by latex agglutination test and Anticyclic citrullinated peptide antibodies (anti CCP) were negative. After getting consent from the patient's mother, a lumber puncture was done under aseptic conditions which revealed normal cerebrospinal fluid (CSF) color and was not under tension, with protein levels of 108 mg/dl (normal values is 15-40 mg/dl) and normal glucose levels. The targets for Epstein-Barr virus, Japanese encephalitis, enterovirus, human cytomegalovirus virus, and herpes simplex viruses 1 and 2 were not detected in CSF by polymerase chain reaction (PCR). CSF cytology and smear analysis showed an acellular pattern. Stains for acid-fast bacilli and fungal elements were negative. Both blood and CSF cultures showed no bacterial growth. PCR for Mycobacterium tubercle bacilli DNA was not detected, and adenosine deaminase (ADA) was 8. The significant ADA level in CSF is more than 10. A complete antibody panel including N- Methyl D-Aspartate Receptors (NMDAR) antibodies and antibodies to Voltage Gated Potassium Channel (VGKC) was planned as part of workup for limbic encephalitis. Plain and contrast-enhanced MRI brain showed leptomeningeal enhancement (Figure 2).

A diagnosis of aseptic meningitis in the pretext of underlying MCTD was made. The patient was started on steroids and antibiotics, following which she showed signs of recovery from day three. Presently, the patient has been on regular follow-up for the past 15 months with a review MRI taken at the end of 12 weeks and was found to be normal. However, due to personal and financial reasons, the patient's attendees were not willing for any other disease-modifying drug treatments.

## Discussion

The patient had one common manifestation in the form of swollen hands, immunological manifestation as high antibody titers, and aseptic meningitis as a characteristic organ involvement for which the diagnosis of MCTD was made. Though the prevalence of aseptic meningitis is reported as 10 to 20% [6] in MCTD, it has been included in characteristic organ involvement along with trigeminal neuralgia in the diagnostic criterion for MCTD [6]. In contrast, the central and peripheral nervous system manifestations in SLE are many and include cerebral infarction, cerebral hemorrhage, cauda equina syndrome, arachnoiditis, myasthenia gravis, optic neuritis, transverse myelitis, etc. Psychosis and seizures, which are more characteristic of lupus, are quite rare in MCTD. The difference in the neurological presentations in lupus as a separate disease and in lupus as a component of MCTD due to the immunological response conferred by the specific autoantibodies to the diseases is a question unanswered. The most commonly detected U1-RNP antibodies are antibodies specifically directed against U1-70K and are found in 75 to 90% of MCTD patients and are reported to appear early [7]. Antibodies to U1-70K, which are apoptotic, are associated with lupus skin diseases and antibodies to oxidatively modified U1-70K have been found to be associated with Raynaud's phenomenon [8]. Patients with U1-RNP have been found to have a higher frequency of human leukocyte antigen (HLA) DR4 allele, and the antibody titers were also found to correlate with disease activity, gaining importance as a prognostic value. However, a few studies have shown that patients with a high titer of anti-U1-RNP rarely develop severe CNS manifestations such as psychosis, seizures, or renal manifestations in the form of diffuse proliferative glomerulonephritis [9]. The titers get lower or disappear during treatment and remission [10]. Steroids and Non-steroidal anti-inflammatory drugs (NSAIDS) commonly used in the management of connective tissue diseases pose a diagnostic dilemma in patients presenting with features of aseptic meningitis as they have also been found to be a cause of meningeal irritation with similar clinical presentation. However, levels of CSF interferon-gamma and interleukin-6 were found to be high in such cases, whereas anti-U1RNP antibodies were not detected in CSF. Drug-induced aseptic meningitis has to be ruled out, as escalating these drugs in routine management of autoimmune disorders can be detrimental. CSF levels of interferon-gamma, interleukin-6, and anti-U1RNP antibodies will help in arriving at a diagnosis. The anti-U1RNP antibodies in CSF are specific only to aseptic meningitis associated with MCTD.

## Conclusions

With a history of low-grade fever, headache, vomiting, and signs of meningeal irritation, subacute meningoencephalitis due to various infectious causes like bacterial, viral, tuberculosis, and non-infectious causes like vasculitis, demyelinating diseases, drug-induced, limbic encephalitis, paraneoplastic, etc. have to be thought of as a differential diagnosis. A detailed past history and examination will narrow down the diagnosis. This patient had a history suggestive of a connective tissue disorder with joint involvement, behavioral disturbances, and a history suggestive of Reynaud’s phenomenon. A diagnosis of MCTD was made taking into account the clinical and immunological findings, and CSF analysis was suggestive of aseptic meningitis. Considered as a subtype of systemic sclerosis in the past, MCTD has recently gained the status of an independent disease due to its characteristic association with conditions such as pulmonary arterial hypertension, aseptic meningitis, and trigeminal neuropathy. MCTD with neurological presentation is very rare and poses great challenges with therapeutic dilemmas. However, the neurological manifestations in lupus are more common, and neuroimaging can reveal small vessel to large vessel and subcortical lesions, meningeal enhancement, etc. Thus, aseptic meningitis with the specific U1-RNP antibodies tilts the diagnosis in favor of MCTD, rules out drug-induced causes, and also adds to the prognostic value. This case highlights the fact that the study of specific antibodies and their modified forms may pave the way for a simple diagnosis and prognostic workup to diagnose MCTD and will help for targeted therapy in the near future.
